# B Cells as a Host of Persistent *Salmonella Typhimurium*

**DOI:** 10.1111/imm.13928

**Published:** 2025-04-14

**Authors:** Alonso D. Cruz‐Cruz, Jocelyn C. Pérez‐Lara, Diana Z. Velázquez, Gabriela Hernández‐Galicia, Vianney Ortiz‐Navarrete

**Affiliations:** ^1^ Departamento de Biomedicina Molecular CINVESTAV Ciudad de México México; ^2^ Programa de Maestría del Departamento de Biología Celular CINVESTAV Ciudad de México México; ^3^ Doctorado en Ciencias de la Producción y Salud animal en la Universidad Nacional Autónoma de México Facultad de Medicina Veterinaria y Zootecnia Ciudad de México México

**Keywords:** B cell, bacterial, flow cytometry

## Abstract

*Salmonella enterica* serovar Typhimurium (*S*. Tm) can colonise different intracellular niches, either actively dividing or remaining dormant to persist. Bacterial persisters are phenotypic variants that temporarily enter a nonreplicative state. This allows them to evade host cell defences and antibiotics, leading to chronic infections. We previously reported that during chronic periods, *Salmonella* remains within B cells in the bone marrow and spleen. However, the dynamics of *Salmonella* replication and the formation of antibiotic tolerance in infected B cells have not been studied. Here we show that B cells are a favourable reservoir for bacterial persistence. In vitro and in vivo experiments identified non‐replicating, persistent *Salmonella* subsets in splenic B cells. These non‐replicative *Salmonella* are tolerant to antibiotics (cefotaxime and ciprofloxacin), while replicative bacteria remain susceptible. Infected mice demonstrated viable, nonreplicative *Salmonella* in spleen B cells, maintaining antibiotic tolerance. Although acid intravacuolar pH and SPI‐2 regulators (SsrA/SsrB) are not necessary for *Salmonella* persistence in B cells, the SehA/B and RelE/B toxin‐antitoxin system facilitates the formation of the persistent phenotype in *Salmonella*. Overall, we show that B cells are a reservoir for nonreplicating, antibiotic‐tolerant *Salmonella*.

## Graphical abstract

B cells: a niche for persistent *Salmonella enterica* serovar Typhimurium (*S.* Tm). In vitro*,* within B cells and in the absence of antibiotics, a persistent population of *S*. Tm emerges, where the SehA/B gene is implicated. Additionally, a replicative population of *S*. Tm is also present. Upon antibiotic administration, approximately 90% of the *S.* Tm population becomes persistent. In vivo, when B cells are infected with *S*. Tm, a population is persistent. Following antibiotic exposure, the replicating *S*. Tm population is eliminated, while the persistent *S*. Tm population retains its phenotype.

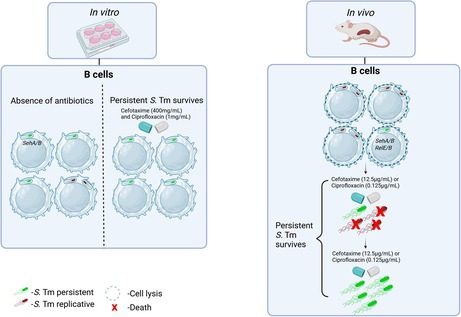

## 1 Introduction

Persistence is the ability of a subset of the bacteria to survive exposure to bactericidal drug concentrations. The induction of the persister phenotype has been proposed to occur within eukaryotic cells in just a few minutes [1], as a response to the environmental stress imposed by the host cell [2].

*Salmonella enterica* serovar Typhimurium (*S*. Tm) can invade different host cells, including epithelial cells, macrophages, dendritic cells, B cells and their B‐cell bone marrow precursors [3, 4, 5]. Within macrophages, *S*. Tm resides in a *Salmonella*‐containing vacuole (SCV), where it secretes SPI‐2 effectors to manipulate intracellular trafficking and signalling pathways, acquiring nutrients and promoting persistence. Toxin–antitoxin (TA) systems have also been considered crucial molecular switches that initiate persistence in bacteria [6, 7, 8].

During B cell infection, *Salmonella* translocates a series of effector proteins through its Type III secretion system (T3SS). Our previous studies have demonstrated that the effector protein SopB blocks Nlrc4 transcription by activating the Akt‐Yap signalling pathway, preventing inflammasome assembly. Thus, infected B cells do not undergo pyroptosis as observed with infected macrophages [9]. The activation of Akt also inhibits autophagy through mTORC1 activation [10]. Thus, both processes contribute to *Salmonella*'s survival within alive B cells.

In mice infected with *S*. Tm, it is capable of survival within splenic B cells and their bone marrow precursors for at least 60 days [4]. However, it has not yet been determined whether *S*. Tm actively proliferates within B cells or if it remains in a dormant state. Hence, in this study, we investigate whether *S*. Tm triggers an intracellular population of persistent bacteria, allowing them to evade the effects of antibiotics and promoting long‐term survival.

## 2 Results

To investigate whether *S*. Tm develops a persistence phenotype in B cells, we employed a fluorescence dilution (FD) assay using wild‐type *S*. Tm carrying the plasmid pFCcGi. This plasmid encodes a constitutively expressed mCherry under the ribosomal promoter *rpsM* and an L‐arabinose‐inducible GFP controlled by the inducible promoter *Pbad*, which is part of the arabinose operon [1]. To induce GFP expression, the bacteria were first cultured in vitro in the presence of arabinose. Upon infecting cells and in the absence of arabinose, the GFP signal gradually decreases with each bacterial cell division. This dilution of green fluorescence enables the tracking of bacterial replication, while mCherry fluorescence identifies the live bacterial population.

**FIGURE 1:**
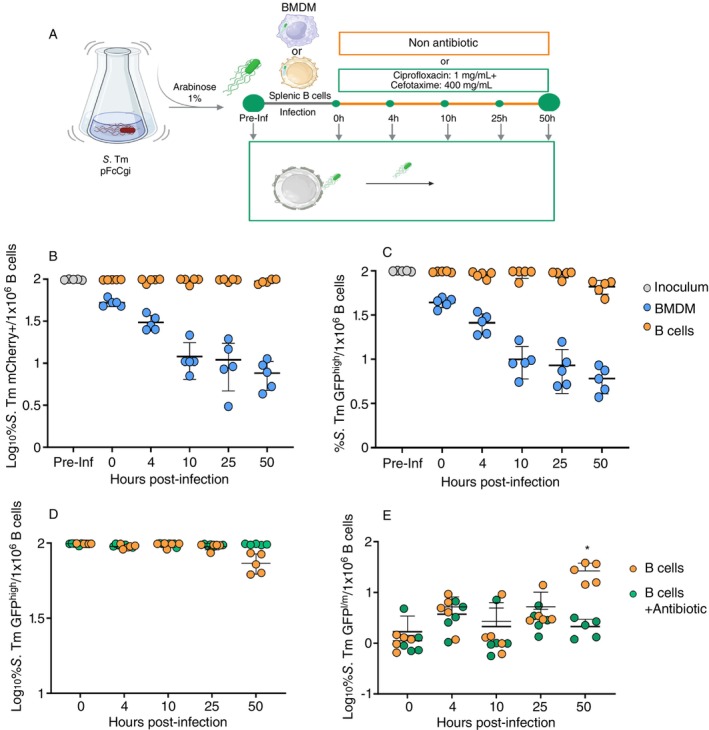
*S*. Tm generates high levels of nonreplicating intracellular subpopulations within B cells. (A) Experimental design: *S*. Tm pFCcGi was cultured in Mg^++^MES medium with 1% arabinose. The excess arabinose was removed, and 1 × 10^6^ B cells or BMDMs from B6 mice were infected at an MOI of 50. The intracellular bacteria were then isolated, and FACS assessed their replicative state and survival. (B) Bacteria kinetics of *S*. Tm mCherry+ frequency or (C) *S*. Tm GFP^high^ frequency recovered from B cells (orange circles), BMDM (blue circles) or before infection (grey circles). (D) Persistent or (E) replicating subpopulations of *S*. Tm in the presence (green) or absence (orange) of antibiotics. The infection process was performed using a multiplicity of infection (MOI) of 50; “pre‐inf” refers to the initial frequency of mCherry+ bacteria in the inoculum. To assess this, a sample of the bacterial culture used for the infection was analysed by flow cytometry. In the graph, the “time 0” point represents the frequency of internalised bacteria after 30 min of infection, corresponding to the interaction period between bacteria and cells. Values are expressed as mean ± SD from five independent experiments. Statistical analysis was performed using two‐way ANOVA followed by Sidak's test, where ****p ≤ 0.001.

We infected purified splenic B cells or nonstimulated BMDM; infected cells were lysed at different time points and quantified intracellular bacteria using flow cytometry. At each indicated time point, 1 × 10^6^ B cells were lysed to recover intracellular bacteria. Following lysis, we gated bacteria based on forward scatter (FSC) and side scatter (SSC) to distinguish them from cellular debris. Within this bacterial gate, we identified mCherry+ viable bacteria, indicating the presence of intracellular *S*. Tm. From the mCherry+ gate, we further selected GFP+ bacteria to differentiate between replicative (GFP‐l/m) and nonreplicative (GFP‐hi) bacterial populations (Figures 1A and S1A). As shown in Figure 1B, BMDM efficiently controlled the intracellular bacteria viability after 10 h postinfection (hpi). In contrast, B cells did not effectively control the *S*. Tm infection, as evidenced by the high average (> 90%) of viable intracellular bacteria at all hpi.

To assess the capacity of *S*. Tm to replicate within B cells, we analysed the GFP fluorescence of viable recovered bacteria. Using FD, we classified recovered intracellular bacteria into nonreplicative (GFP^high^) and replicative (GFP^med^ + GFP^low^) categories (Figure S1B). We found a higher frequency of nonreplicative bacteria in B cells since early hpi, in contrast with the rate observed in BMDM. The percentages of nonreplicative bacteria were maintained until 25 h after infection, but at 50 hpi, we observed a proliferative subset in B cells. In contrast, in BMDM, only half of the bacteria were nonreplicative at the onset of infection, and this percentage progressively decreased over time (Figure 1C). These results demonstrated that *S*. Tm maintains a high index of viable but nonreplicative bacteria in B cells and corroborate that B cells are less effective at controlling intracellular bacteria compared to macrophages.

To corroborate the existence of a nonreplicative phenotype, we cultured *S*. Tm infected B cells with two cell‐permeable antibiotics, cefotaxime and ciprofloxacin, and subsequently evaluated the antibiotic tolerance of intracellular bacteria. Our observations showed that even in the presence of antibiotics, we observed the maintenance of *S*. Tm GFP^high^ (Figure 1D). Additionally, we showed in Figure 1E the frequency of replicating bacteria, expressed as the percentage of GFP l/m. As expected, the replicative bacteria at 50 hpi were not detected in the presence of antibiotics (Figure 1E). These results indicate that *S*. Tm predominantly resides in a nonproliferative state within B cells, which became tolerant to antibiotic treatment.

## 3 *S*. Tm Internalisation by Splenic B Cells Promotes Nonreplicating Population Formation in Vivo

**FIGURE 2:**
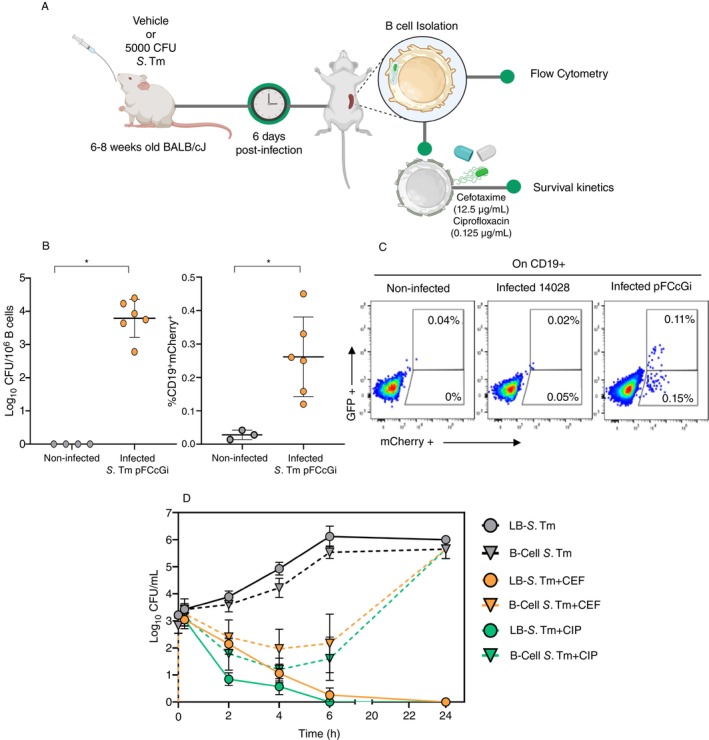
*S*. Tm internalisation by splenic B cells promotes nonreplicating population formation in a diseased host. (A) Experimental design: BALB/cJ mice were infected via gavage with 5000 CFU of *S*. Tm pFCcGi, and spleen B cells were isolated 6 days postinfection. Flow cytometry was performed to identify *S*. Tm intracellular. Subsequently, the intracellular bacteria were recovered from lysed B cells. Then they cultured in the absence or presence of cefotaxime (12.5 μg/mL) or ciprofloxacin (0.125 μg/mL) (B) CFUs recovered from B cells infected 6 days postinfection. Statistical analysis was performed using Student *t* test, where *p < 0.05. (C) Right: Frequency of intracellular *S*. Tm mCherry+; left: Representative dot plots of mCherry+ and GFP+ subsets in CD19+ B cells. Statistical analysis was performed using Student *t* test, where *p < 0.05. (D) Cefotaxime (CEF) and Ciprofloxacin (CIP) survival kinetics of *S*. Tm from splenic B cells (B cell‐*Salmonella* T.) or *S*. Tm previously cultured in Luria Bertani medium (LB‐*Salmonella* T.).

The persistent phenotype has been well established in vitro; however, their identification in vivo remains poorly explored. To investigate whether nonreplicating bacteria could be detected in infected mice, we conducted an experiment where mice were infected via gavage with 5 x 10^3^ CFU of *S*. Tm pFCcGi. Then, we examined the presence of the intracellular bacteria at 6 days postinfection (dpi) (Figures 2A and S1C). Our data showed that splenic B cells carry viable bacteria with a burden ranging from 10^2^ to 10^**4**^ CFU per 1 × 10^6^ B cells (Figure 2B). As shown in Figure 2C, we identified a population of replicating and nonreplicating *S*. Tm within B cells isolated from infected mice, representing 0.26% (0.15% of replicating and 0.11% of nonreplicating). These results resemble our in vitro data (Figure 1C).

To examine the antibiotic tolerance of the in vivo generated nonreplicative subpopulations, we accomplish an ex vivo antibiotic tolerance assay. Intracellular bacteria were recovered from splenic B cells lysed with 0.1% Triton and subsequently cultured in the presence or absence of cefotaxime or ciprofloxacin. As a control, *S*. Tm cultured in rich‐nutrient media LB medium was subjected to the same culture conditions. As shown in Figure 2D, the intracellular nonreplicative *S*. Tm (B‐cell *S*. Tm) exhibited the same growth capabilities as those observed with the bacteria always cultured in rich‐nutrient media LB (LB‐*S*.Tm). When LB‐*S*. Tm was cultured in the presence of antibiotics, no CFU was recovered at 6 hpi. On the contrary, the B‐cell *S*. Tm showed susceptibility to the antibiotics, but after 6 hpi, a population began to grow, reaching the same magnitude as that cultured in the absence of antibiotics. These results demonstrate that, in fact, during in vivo infection, B cells have the conditions for generating nonreplicative bacteria with antibiotic tolerance.

## 4 The Induction of the Persistent Phenotype in B Cells Is Independent of Acid Intravacuolar pH and *S*. Tm Pathogenicity Island 2 (SPI‐2)

**FIGURE 3:**
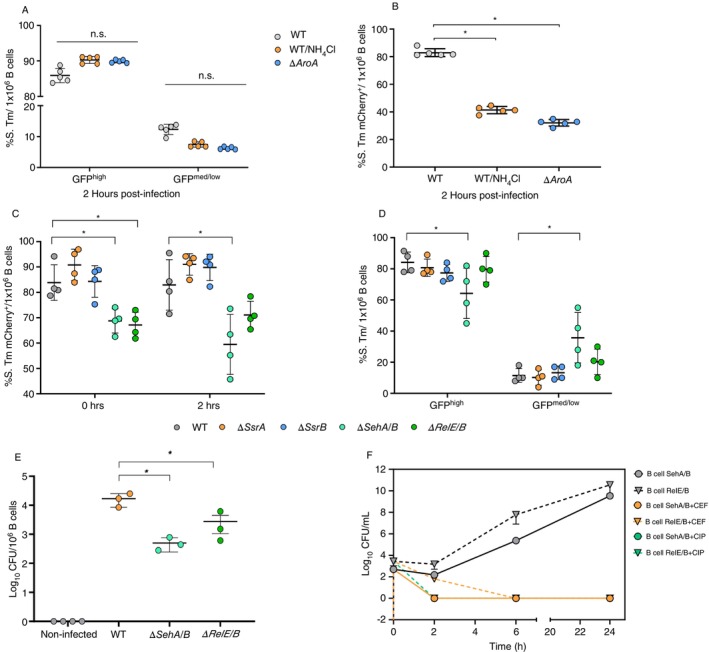
The SehA/B toxin‐antitoxin system facilitates the formation of the persistent phenotype in *S*. Tm. (A and B). Frequency of *S*. Tm GFP^high^ and GFP^med/low^ (A) or frequency of *S*. Tm mCherry+ (B) when B cells from B6 mice were infected. Data represent the phenotype of *S*. Tm WT (grey) or ∆*Aroa* (blue), after 2 h postinfection. B cells from B6 mice were pretreated with NH_4_Cl prior infection with *S*. Tm WT (orange). (C and D) Frequency *S*. Tm survival (mCherry^+^) (C) and nonreplicating (mCherry^+^GFP^high^) and replicating (mCherry^+^GFP^med/low^) bacteria of WT or *S*. Tm mutant strains (D). (E) CFU recovery from B cells infected in vivo with ∆*SehA/B* and ∆*RelE/B* 6 dpi in BalB/cJ mice. (F) Antibiotic tolerance assay of *S*. Tm ∆*SehA/B* and ∆*RelE/B* mutants, recovered from splenic B cells isolated from infected mice at 6 days postinfection, then cultured in LB broth in the presence of cefotaxime (CEF, 12.5 μg/mL) or ciprofloxacin (CIP, 0.125 μg/mL). CFUs were enumerated at 0, 2 and 6 h to evaluate bacterial survival. Statistical analysis was performed using one‐way ANOVA followed by Tukey's post hoc test (*p < 0.05).

To analyse the role of the intravacuolar pH in the generation of nonreplicative *S*. Tm, B cells pretreated with the SCV‐alkalising agent NH_4_Cl were infected with *S*. Tm. Despite the treatment, we did not observe a reduction in intracellular persistence or replicative subpopulations of *S*. Tm (Figure 3A).

It has been demonstrated that a metabolically attenuated strain of *S*. Tm (*aroA* ^−^) shows decreased replicative capabilities within macrophages [11, 12]. Therefore, we also analysed whether the aroA strain behaves as the virulent strain within B cells. Remarkably, the aroA generated both the nonreplicative and the replicative populations (Figure 3B), and this occurrence was independent of the intravacuolar pH for both strains.

To elucidate the participation of SPI‐2 in generating the nonreplicative population, we used *S*. Tm mutants lacking the SPI‐2 regulators and sensors, ΔSsrA or ΔSsrB. Our results showed that both genes are dispensable for early intracellular survival, as both *S*. Tm populations were observed (Figure 3C). These results demonstrate that the regulators SsrA/SsrB are not required to induce nonreplicative *S*. Tm within B cells.

## 5 TA systems sehA/B contributes to the Formation of the Persistent Phenotype During B‐Cell Infection

While the TA system has previously been proposed as one of the crucial mechanisms for inducing intracellular persistence in BMDM [1], its role in B cells remains unknown. Here, we tested two *S*. Tm TA systems, SehA/B and RelE/B. We identified both TA systems as important to promote the infection of B cells, as a lower frequency of bacteria was observed at the beginning of the infection, but bacteria remained viable, and the nonreplicative and replicative populations were generated. However, with the SehA/B mutant, a lower frequency of the nonreplicative population was observed; consequently, a higher percentage of the replicative population was noted (Figure 3C,D). Then we aimed to determine if the TA systems (*SehA/B* and *RelE/B*) play comparable roles in bacterial persistence under antibiotic pressure in an in vivo infection model. The *SehA/B* and *RelE/B* mutants showed significantly reduced CFU recovery from B cells at 6 days postinfection compared to the wild‐type strain, indicating that these TA systems are critical for bacterial infection in B cells (Figure 3E). We observed that SehA/B bacteria recovered from B lymphocytes at 6 days postinfection were eliminated within the first 2 h when cultured in the presence of cefotaxime and ciprofloxacin. In contrast, the RelE/B mutant exhibited partial survival during the first 2 h of antibiotic treatment with cefotaxime. However, by 6 h posttreatment, the mutant was also completely eliminated, similar to the wild‐type strain (Figure 3F). Unlike what was observed in vitro, both systems (SehA/B and RelE/B) are required to induce antibiotic tolerance in infected B cells. Despite these findings, the exact mechanism by which *S*. Tm establishes a persistent phenotype in B cells remains to be elucidated.

## 6 Discussion

Brucella, Francisella, Mycobacterium and Listeria can infect B cells, using various mechanisms to survive within them [13]. Here, we show that *S*. Tm remains nonproliferative in B cells, a state enhanced by antibiotics and partially established by the SehA/B TA system, a mechanism previously described as a persistence inductor in macrophages [1, 6].

We showed that macrophages gradually eliminate nonreplicating (GFP^high^) bacteria and control the infection in vitro, compared with B cells which remain infected. As we show in Figure S1D, CFU recovered from B cells declined consistently over time. From 0 to 25 h, CFU were recovered, but at 50 h, no colony grew. However, by flow cytometry, we observed viable bacteria based on mCherry expression. This contradictory data may be because, at 50 h postinfection, we observed some *Salmonella* replication, and due to the absence of ampicillin in the medium, there is probably insufficient plasmid segregation, which can lead to a decrease in plasmid copy number. The decreasing bacteria amount and the reduction in plasmid copy number may result in underestimating the number of plasmid‐containing *Salmonella* when using the plate count (PC) assay. In this context, *Lactobacillus reuteri* studies demonstrated that the mCherry expression system remained stable over 100 generations, indicating robust expression without significant degradation over extended periods, even without selective pressure [14]. Thus, flow cytometry offers a more robust approach to detecting a low number of bacteria with fewer plasmid copies, which may not be recoverable via PC assay in the presence of ampicillin.

In vivo, we identified viable bacteria in B cells 6 days after infection; likewise, a high proportion conserves a nonproliferative status. As shown in Figure 2C, we identified a population of replicating and nonreplicating *S*. Tm within B cells isolated from infected mice, representing approximately 0.26% (0.15% of replicating and 0.11% of nonreplicating). Our in vitro results suggest that *S*. Tm undergoes an initial phase of moderate proliferation around 50 h postinfection. In contrast, our in vivo observations indicate that this phase is followed by a period of population stabilisation, during which a subset of nonreplicating bacteria is maintained within B cells at 6 dpi.

It has previously been reported that *S*. Tm enhances its survival within B cells through the SopB protein, which activates the PI3K/Akt pathway and inflammasome formation inhibition due to cytosolic retention of YAP phosphorylated. As a result, SopB allows *Salmonella* to prevent pyroptosis in B lymphocytes, supporting its survival, slow elimination and probably persistence establishment [9, 13, 15].

Our results demonstrate that antibiotic treatment favours the persistence phenotype (GFP^high^) maintenance, probably due to the elimination of actively proliferating bacteria, induction of long‐lasting persistent genes, or working as a selective pressure to avoid persistence switch. Likewise, we demonstrate that recovery bacteria from infected mice are heterogeneous, and we detect at least two subpopulations: one that is nontolerant and another that is tolerant to treatment, capable of replicating under nutritive conditions. The latter is probably the persistent bacteria, as was demonstrated by Helaine and cols in *S*. Tm‐infected macrophages [1]. It is now well established that intracellular bacterial populations are heterogeneous and may exhibit different levels of susceptibility to antibiotics [2]. The induction of bacterial persistence is enhanced under various stress conditions, including host‐induced stress and exposure to bactericidal antibiotics. In our study, we apply two distinct stress factors: the stress experienced by bacteria within the B cell host environment in the mouse organism and the stress imposed by antibiotic treatment. We hypothesise that bacteria become more vulnerable to mutation‐driven antibiotic resistance in this persistent state, particularly if they possess error‐prone DNA repair mechanisms. This heightened susceptibility to mutations may contribute to the development of antibiotic resistance during persistent infections.

In this model, the persistence induction was independent of SsrA/SsrB expression, a two‐component regulatory system SsrA/SsrB in *S*. Tm that controls the expression of SPI‐2. However, the role of this system (SsrA/SsrB) in persistence induction depends on the cellular type and antibiotics used to characterise persistent bacteria.

The larger persister population in B cells likely reflects differences in how these cells interact with *S*. Tm. Unlike macrophages, which are professional phagocytes equipped with potent bactericidal mechanisms (e.g., reactive oxygen species, nitric oxide production and acidic vacuoles), B cells provide a less hostile intracellular environment. This is supported by previous findings that *S*. Tm within B cells is housed in SCVs exhibiting late endosomal‐lysosomal compartment characteristics. At the same time, the SCV within macrophages remodels into a nonconventional late endosomal lysosomal compartment [3]. These differences likely create conditions in B cells that are more conducive to the survival and persistence of a larger fraction of the bacterial population.

In macrophages, *S*. Tm persistence heavily relies on the activity of the *Salmonella* Pathogenicity Island‐2 (SPI‐2) effectors, which modify the SCV environment to evade host defences. Our data indicate that SPI‐2 regulators (SsrA/SsrB) are dispensable for persistence in B cells, suggesting that *S*. Tm employs alternative mechanisms in this cell type. Alternative mechanisms may involve the modulation of host signalling pathways (e.g., PI3K/Akt, mTORC1) and inhibition of autophagy, as demonstrated previously, which allow B cells to survive for chronic periods.

Our data suggests that in B cells, SCV's pH does not impact persistence establishment, implying that other factors, such as nutrient deprivation and osmolarity, might be involved [2, 12, 13].

We studied the TA systems sehA/B and RelE/B. TA systems can downregulate essential functions, triggering a persister state that allows cells to survive unfavourable conditions without acquiring mutations. This slowing of essential metabolic processes can lead to tolerance to antimicrobials and recalcitrant infections. Our data demonstrate that while the SehA/B system is sufficient to promote *S*. Tm persistence in B cells under in vitro conditions, the RelE/B system does not appear to play a significant role. However, in vivo experiments revealed that both SehA/B and RelE/B systems are necessary to induce antibiotic tolerance, indicating that the in vivo environment introduces additional factors that influence bacterial persistence. The exact mechanism of persistence formation in B cells remains unknown.

We previously reported that *S*. Tm inhibits autophagy in B cells because of SopB expression, indicating that nutrient deficiency could affect its proliferation through this mechanism [10]. Our results rule out the restriction of the synthesis of aromatic amino acids, as seen in the *S*. Tm *aroA* ^*−*^ strain. This study is the first to identify B cells as a reservoir and inducer of *S*. Tm persistence, which may explain antibiotic treatment failures and contribute to the evolution of virulence and asymptomatic carriers.

While our findings highlight splenic CD19+ B cells as an important reservoir for persistent *S. Tm*, they are not the only host cells capable of supporting bacterial persistence. However, other immune cells including dendritic cells, neutrophils or T cells might also have become a reservoir for *S*. Tm, but this has not been evaluated yet. Each cell type provides a distinct intracellular environment that shapes the dynamics of bacterial survival, replication and persistence. Understanding these diverse niches is crucial for comprehensively addressing chronic infection, as *S. Tm* likely utilises a combination of strategies to persist across multiple host compartments.

## 7 Materials and Methods

### 7.1 Bacterial Strain and Growth Conditions

*S*. Tm 14028 (*S*. Tm; ATCC) was transformed with the plasmid pFCcGi (Addgene plasmid #59324; http://n2t.net/addgene:59324; RRID), which constitutively expresses mCherry and GFP expression is inducible by arabinose. The transformed bacteria were cultured in LB broth (Luria Bertani) with ampicillin for 12 h, followed by secondary culture in minimal MES^Mg+^ medium [16]. The *S*. Tm mutant (ΔSehA/B and ΔRelE/B) strain was kindly donated by Dr. Miguel Angel De la Cruz Villegas (PhD Full Research Professor B T. C. at Benemerita Universidad Autonoma de Puebla). The *S*. Tm mutants ΔSsrA and ΔSsrB strain was generated using the λ‐red system as was described elsewhere [16, 17] (the λ‐red system was kindly provided by William Navarre's laboratory at Toronto University).

### 7.2 Mice

BALB/cJ and C57BL/6 (B6) mice aged 6–8 weeks bred in the Animal Production Unit of the Center for Research and Advanced Studies (CINVESTAV), Mexico City, Mexico, were used.

### 7.3 Cell Cultures and Lines

B cells from B6 mice were purified from the spleen according to the manufacturer's instructions (Miltenyi Biotec 130.090.862). To obtain bone marrow‐derived macrophages (BMDMs), bone marrow precursors were cultured with DMEM medium supplemented with 10% FBS, antibiotic/antimycotic, and L929 supernatant.

### 7.4 In Vitro Infections

BMDMs or purified B cells from the spleen of B6 mice were infected at an MOI of 50. Bacteria are allowed to infect B cells for 30 min. Following this infection period, extracellular bacteria are removed by washing the cells with gentamicin‐containing PBS to eliminate any loosely attached bacteria. To assess bacterial survival and replication within macrophages or B cells, the infected cells were then incubated in a medium containing gentamicin (80 mg/mL). This was maintained throughout the experimental duration (4, 10, 25 and 50 h). Then, cells were lysed at different postinfection times (4, 10, 25 and 50 h), and bacterial phenotype was analysed by flow cytometry (CytoFLEX LX).

### 7.5 In Vivo Infections

Infections were performed orally with 5000 CFU. Six days postinfection, splenocytes were isolated under sterile conditions, and B cells were purified (previously described) for CFU recovery and flow cytometry analysis.

### 7.6 Antibiotic Tolerance Assay

B cells (1 × 10^6^) from spleens of infected BALB/cJ mice were isolated, lysed and then recovered bacteria were cultured either in the presence of cefotaxime (12.5 μg/mL), ciprofloxacin (0.125 μg/mL) or without antibiotics. Bacteria‐grown conditions in LB broth were similarly treated. CFU were recovered after 2, 4, 6 and 24 h of antibiotic exposure.

### 7.7 Data Analysis

Significance determined by ANOVA and indicated post hoc multiple comparison tests, analysed on GraphPad Prism. *p* < 0.05 is considered significant. Flow cytometry data were analysed using Flow‐Jo v.10.

## Author Contributions

A.D.C.‐C., J.C.P.‐L. and V.O.‐N. conceived and designed the experimental protocols. J.C.P.‐L., D.Z.V. and A.D.C.‐C. performed the experiments, analysed the data, drafted the manuscript and prepared the figures. G.H.‐G. and V.O.‐N. edited and revised the manuscript. V.O.‐N. funded the acquisition. All authors have read and agreed to the published version of the manuscript.

## Ethics Statement

All animal experiments were performed in strict accordance with the recommendations of the Official Mexican Norm NOM‐062‐ZOO‐1999. The protocol was approved by the Committee for Laboratory Animal Care of the Center of Research and Advanced Studies (CICUAL‐CINVESTAV) under approval number 0078–14.

## Conflicts of Interest

The authors declare no conflicts of interest.

## Supporting information


**Figure S1.** *S*. Tm generates replicating and nonreplicating subpopulations.
(A) Analysis of intracellular bacterial subpopulations of *S*. Tm pFCcGi in splenic B cells or BMDMs using flow cytometry. The dot plot shows viable bacteria (mCherry+) analysed based on their GFP expression and classified according to their replicative status into replicators and non‐replicators (persistent). (B) Image obtained by confocal microscopy: bacteria grown in vitro in minimal medium (Mg++‐MES+arabinose 1%); nonreplicating or persistent bacteria indicated with green arrow/GFP^high^ and replicating bacteria with yellow arrow GFP^low^ and red arrow GFP^neg^. (C) Gated CD19+ B cells were analysed for *Salmonella typhimurium* (*S. Tm*) infection by assessing mCherry and GFP fluorescence. (D) CFU recovered from infected B cells at different time points, as shown in Figure 1.

## Data Availability

The data that support the findings of this study are available from the corresponding author upon reasonable request.
