# Supplementary figures and images for: B Cells as a Host of Persistent Salmonella Typhimurium
Source: Immunology. 2025 Apr 14;175(3):292–9. doi: 10.1111/imm.13928 (PMC12130668; doi:10.1111/imm.13928)

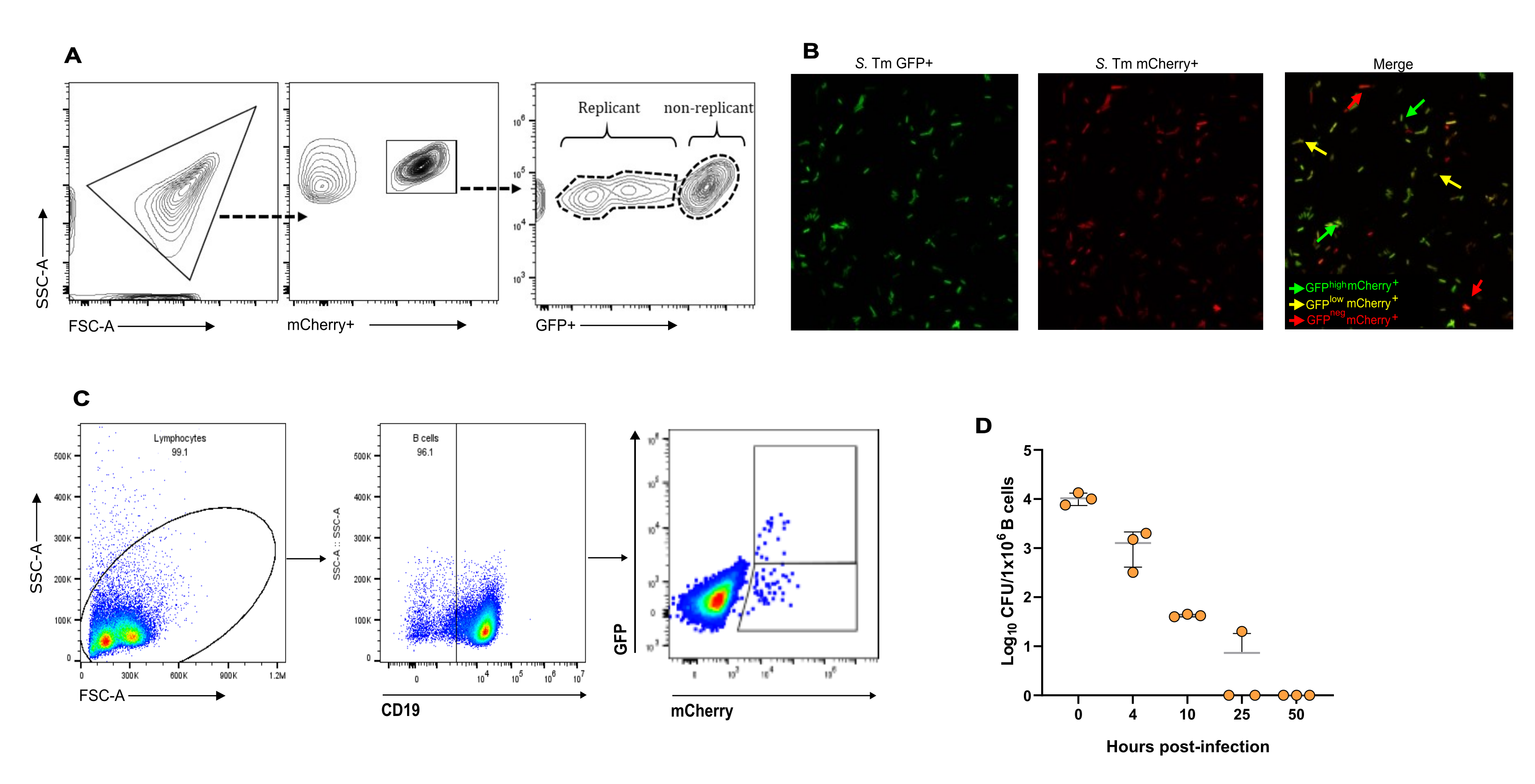

Supplement: Supplementary file 1 — Figure S1. S. Tm generates replicating and nonreplicating subpopulations. (A) Analysis of intracellular bacterial subpopulations of S. Tm pFCcGi in splenic B cells or BMDMs using flow cytometry. The dot plot shows viable bacteria (mCherry+) analysed based on their GFP expression and classified according to their replicative status into replicators and non‐replicators (persistent). (B) Image obtained by confocal microscopy: bacteria grown in vitro in minimal medium (Mg++‐MES+arabinose 1%); nonreplicating or persistent bacteria indicated with green arrow/GFPhigh and replicating bacteria with yellow arrow GFPlow and red arrow GFPneg. (C) Gated CD19+ B cells were analysed for Salmonella typhimurium (S. Tm) infection by assessing mCherry and GFP fluorescence. (D) CFU recovered from infected B cells at different time points, as shown in Figure 1. [file IMM-175-292-s001.tiff]
